# Interactions between Intestinal Microflora/Probiotics and the Immune System

**DOI:** 10.1155/2019/6764919

**Published:** 2019-11-20

**Authors:** Chen-xing Zhang, Hui-yu Wang, Tong-xin Chen

**Affiliations:** ^1^Department of Rheumatology and Immunology, Shanghai Children's Medical Center Affiliated to Shanghai Jiao Tong University School of Medicine, Shanghai, China; ^2^Department of Nephrology, Shanghai Children's Medical Center Affiliated to Shanghai Jiao Tong University School of Medicine, Shanghai, China; ^3^Division of Immunology, Institute of Pediatric Translational Medicine, Shanghai Jiao Tong University, Shanghai, China; ^4^Institute of Physiological Chemistry and Pathobiochemistry, University of Muenster, Muenster, Germany

## Abstract

The digestive tract is home to millions of microorganisms and is the main and most important part of bacterial colonization. On one hand, the abundant bacterial community in intestinal tissues may pose potential health challenges such as inflammation and sepsis in cases of opportunistic invasion. Thus, the immune system has evolved and adapted to maintain the symbiotic relationship between host and microbiota. On the other hand, the intestinal microflora also exerts an immunoregulatory function to maintain host immune homeostasis, which cannot be neglected. In addition, the interaction of either microbiota or probiotics with immune system in regard to therapeutic applications is an area of great interest, and novel therapeutic strategies remain to be investigated. The review will elucidate interactions between intestinal microflora/probiotics and the immune system as well as novel therapeutic strategies.

## 1. Intestinal Immune System

Gut associated lymphoid tissue (GALT) is composed of the epithelium, lamina propria, and muscular layer [1]. Enterocytes constitute most of the intestinal epithelial cells and are able to absorb sugar, amino acid, and many other nutrients. Some enterocytes express Toll-like receptors (TLRs) and will secrete a series of proinflammatory chemokines (IL-8), cytokines (IL-1, IL-6, IL-7, IL-11, and TNF), and growth factors (SCF and G-CSF) when encountering with pathogens or toxins. These molecules will recruit peripheral neutrophils and mast cells to intestinal subepithelial regions and accelerate activation and differentiation of local lymphocytes. For instance, IL-7 and SCF secreted by intestinal epithelial cells can act synergistically to activate *γδ* intestinal intraepithelial lymphocytes (iIELs). Then, activated *γδ*–iIEL can also secrete cytokines and chemokines to activate *αβ*–iIEL, thus initiating a more robust adaptive immune response [2–4]. Between intestinal epithelial cells are enteroendocrine cells, paneth cells, and goblet cells. When a pathogen invades the body, paneth cells release certain antibacterial molecules such as defensins into villi in the small intestine lumen while goblet cells secrete mucus to the intestinal surface, which is helpful for maintaining the intestinal barrier [5, 6]. Intraepithelial *αβ*T and *γδ*T lymphocytes, NK cells, and NKT cells can also be gathered among intestinal epithelial cells. Intestinal intraepithelial lymphocytes (iIELs) are a unique cluster of cells which reside in intestinal mucosal epithelium and have two different cell sources. Approximately 40 percent of iIELs are thymus-dependent *αβ* T cells and their phenotype is similar to peripheral T cells. About 60 percent of iIELs are thymus-independent *γδ* T cells. *γδ* T cells are innate immune cells with strong cytotoxicity as well as the capacity to secrete various cytokines. Therefore, iIEL plays a vital role in immunosurveillance and cell-mediated mucosal immunity [7–9].

Lamina propria contains a large number of macrophages and neutrophils as well as a small number of NKT cells, mast cells, and immature dendritic cells. A certain number of mature *αβ* T cells and B cells as well as few *γδ* T cells also reside in the lamina propria [10, 11]. Lymphocytes in the lamina propria usually congregate together to form intestinal follicle, which contains germinal centers populated by B cells and follicular dendritic cells, topped by immature dendritic cells, macrophages, CD4^+^T cells, and mature B cells [12, 13]. Located in one side of intestinal follicle that is close to the intestinal luminal are specialized phagocytic cells named M cells, which can transport antigens across the epithelium to the side of basement membrane via transcytosis. Consequently, the antigens interact with the local immune cells and initiate mucosal immune responses where B cells differentiate into IgA secreting plasma cells [14–16]. The elements of intestinal mucosal immunity are summarized in Table 1.

The intestine is a unique organ which is in close contact with microorganisms. Most microbes are destroyed and killed by the harsh gastric acid environment, but a few can still make it through the intestine. The intestinal surface is covered with a large number of finger-like projections called microvilli (also named brush border), whose primary function is the absorption of nutrients. Brush border is wrapped up by a molecule called glycocalyx [17]. Since glycocalyx is a negatively charged and mucoid glycoprotein complex, microvilli could prevent the invasion of pathogenic bacteria. Besides, apical tight junctions of intestinal epithelial cells also ensure that pathogens do not pass through the intestine [18]. A vast population of immune cells reside within these and the underlying structures. As the most crucial intestinal sentinels, Peyer's patches are composed of B-cell follicles, interfollicular regions, macrophages, and dendritic cells [19]. A key function of Peyer's patch is sampling of particulate antigens, mostly bacteria and food through a specialized phagocytic cells called M cells, which can transport material from the lumen to subepithelial dome [20]. Then, local dendritic cells are able to sample antigens and present them to immune effector cells [21]. Nevertheless, intestinal tolerance is mainly mediated by CD4+ Treg cells in the context of uptake of food antigens. These Treg cells secrete IL-10 and TGF-*β* which exerts suppressive effects on immune cells within the lamina propria. However, a breakdown in the process of immune hemostasis will lead to gut pathology such as food allergy and inflammatory bowel disease [22, 23]. Intestinal barriers including mucin, antimicrobial peptides, and secretory IgA prevent the direct contact between the microorganisms and gut epithelial layer. Barrier destructions can contribute to bacteria influx, activation of epithelium, and inflammatory responses [24]. Proinflammatory antigen-presenting macrophages and dendritic cells are activated and release inflammatory cytokines such as IL-6, IL-12, and IL-23. Th1 and Th17 effector T-cell subsets are polarized and produce inflammatory cytokines such as TNF-*α*, IFN-*γ*, and IL-17 [25]. In addition, neutrophils are recruited and undergo dramatic form of cell destruction called NETosis, with the production of neutrophil extracellular traps (NETs) and tissue injuries [26].

## 2. Intestinal Microflora and Probiotics

There are a large number of microorganisms in the intestine, which are mainly distributed in the colon. It is estimated that over 40 trillion bacteria (including Archaebacteria) inhabit in the colon of adults, with a small proportion of fungus and Protista. In general, each individual carries an average of 600,000 intestinal microbial genes [27, 28]. In terms of bacterial strains, there is a distinct diversity among individuals. Each individual has his unique intestinal microflora, which is determined by host genotype, initial colonization through vertical transmission at birth, and dietary habits [29–32]. In healthy adults, the composition of bacterial flora in feces is stable regardless of time. Bacteroidetes and Firmicutes are two main bacteria in human intestinal ecosystem, accounting for over 90 percent of all microorganisms. The remains are Actinobacteria, Proteobacteria, Verrucomicrobia, and Fusobacteria [33, 34]. Probiotics are microorganisms that may be beneficial to health when consumed in adequate amounts [35]. Lactobacillus and Bifidobacteria are most commonly applied probiotics in clinical practice. Yeast *Saccharomyces boulardii* and *Bacillus* species are also widely used [36, 37]. The function of probiotics is closely related to the species of microorganisms that colonize within the intestine. The interaction between probiotics and host cells as well as intestinal flora is a key factor which influences the host health. Probiotics have an impact on intestinal ecosystem by regulating gut mucosal immunity, by having interactions with commensal microflora or potentially harmful pathogens, by producing metabolites (such as short-chain fatty acids and bile acids), and by acting on host cells through signaling pathways (Table 2). These mechanisms can contribute to the inhibition and elimination of potential pathogens, improvement of intestinal microenvironment, strengthening the intestinal barrier, attenuation of inflammation, and enhancement of antigen-specific immune response [38, 39].

Disturbed intestinal immune niche is a contributory cause for the digestive diseases such as inflammatory bowel disease (IBD), functional dyspepsia, gastroesophageal reflux disease, and nonalcoholic fatty liver disease. IBD patients are characterized by an increase in potentially aggressive gut microbial strains as well as decreased regulatory species [40–42]. Aggressive gut microbial strains activate inflammatory response by inducing Th1 and Th17 effector cells while decreased regulatory species inhibit the generation and function of regulatory cells including regulatory T cells (Treg), B cells (Breg), macrophages, dendritic cells (DCs), and innate lymphoid cells (ILCs). This has further resulted in elevated levels of TNF-*α* and inflammasome and reduced levels of IL-10, TGF-*β*, and IL-35 [43]. Therefore, dysbiosis of the intestinal flora has contributed to dysfunctional immune system and the chronic inflammation in IBD.

## 3. Immune Regulation by Microflora and Probiotics

### 3.1. Promoting the Balance of Th1, Th2, Th17, and Treg Cells

Actually, intestinal microorganism can elicit diverse signals and induce CD4+T-cell differentiation. Invasive bacteria such as ectopic colonization of *Klebsiella* species can induce DCs phagocytosis and release of proinflammatory cytokines (IL-6, IL-12, and TNF), which is closely associated with Th1 polarization. *Bacteroides fragilis* is a kind of symbiotic anaerobic bacteria which colonizes in human lower digestive tract. Polysaccharide A (PSA) in its outer membrane can be recognized by T-cell surface molecule TLR2, which induces differentiation of CD4+T cells into Treg cells. Here, the Treg cells secrete molecules such as IL-10 and TGF-*β* which exert a suppressive action on immune cells. Actually, it has been demonstrated that administration of PSA or intestinal *Bacteroides fragilis* colonization can prevent intestinal inflammatory diseases in mice models [44–46]. In addition, segmented filamentous bacteria can be presented to T cells by dendritic cells and contribute to the synthesis of Th17 cells in lamina propria of small intestine, thus playing a vital role in antibacterial immune response [47, 48]. Parasites, for instance, *Heligmosomoides polygyrus*, can contribute to a Th2 immune response. The parasite can bind to tuft cells and secret high amounts of IL-25, which then acts upon dendritic cells. Dendritic cells produce IL-4 and TGF-*β* and induce CD4+ T differentiation into Th2 subset, with upregulated levels of IL-4 and GATA3 transcription factor. The immunomodulatory effects of various probiotics are listed in Table 3.

### 3.2. Regulation of Intestinal Related Gene Expression

Previous reports have demonstrated that expression of multiple intestinal genes is regulated by probiotics. For instance, *Escherichia coli* and *Lactobacillus rhamnosus* can upregulate mucin expression in intestinal cells to enhance intestinal mucosal barrier. Probiotics can also regulate gene expression of enterocytes and dendritic cells. It has been demonstrated that probiotic VSL#3 in certain concentrations (10^7^ organisms/mL) could alter the DC phenotypes by the upregulation of costimulatory molecule (CD80, CD86, and CD40) expression [49].

### 3.3. Regulation of Immune Response through Microbial Metabolites

Probiotics can produce a series of metabolites by digesting different foods and impact the immune response within the body.

#### 3.3.1. Short-Chain Fatty Acids

Short-chain fatty acid (SCFA) is fatty acid with carbon chain length of 1–6 carbon atoms. It is produced through fermentation of fibres by probiotics. Intestinal SCFA mainly includes acetate, propionate, and butyrate. SCFA can exert its immunoregulatory function as both extracellular and intracellular signaling molecules [50, 51]. Extracellularly, SCFA can act as ligands for cell surface G protein coupled receptors such as GPR41, GPR43, and GPR109a and regulate immune function indirectly. SCFA can bind to GPR43 in the surface of neutrophils and eosinophils to alleviate intestinal inflammation. GPR109a, which is expressed in colon epithelial cells and innate immune cells, can specifically bind to butyrate and induce differentiation of Treg cells [52, 53]. Intracellularly, SCFA can inhibit histone deacetylases (HDAC) and regulate gene transcription to exert immunoregulatory functions. For example, SCFA can promote acetylation of FoxP3 and synthesis of colon FoxP3+Treg cells to enhance their immunosuppressive function. Butyrate can suppress HDAC activity of macrophages in intestinal lamina propria and inhibit their secretion of inflammatory mediators such as nitric oxide, IL-6, and IL-12 [54, 55]. In addition, SCFA can also promote Tfh-cell production, B-cell differentiation, and antibody synthesis, as evidenced by latest reports [56].

SCFA also plays a crucial role in homing of T cells. Retinol, the main component of vitamin A, can be oxidized into retinaldehyde by retinol dehydrogenase. Retinal can be further oxidized to retinoic acid (RA) in vivo through an enzyme called Aldh1a. SCFA, the metabolites of probiotics, increases the activity of Aldh1a and promotes the conversion of intestine absorbed vitamin A into RA. Dendritic cells in intestinal Peyer's patch (PP) and mesenteric lymph nodes (MLN) express Aldh1a1 and Aldh1a2, respectively, and therefore produce RA locally. When an antigen is presented to T cells by CD103+ dendritic cells in MLD, the local RA induces expression of *α*4 in T-cell surfaces, which then binds with *β*7 to form *α*4*β*7 integrin. The *α*4*β*7 integrin can combine with MadCAM-1 molecule of high endothelial vein (HEV) surface. Meanwhile, RA also induces CCR9 expression in T-cell surface, which binds to CCL25 in intestinal epithelial cells [57, 58]. Therefore, probiotics can promote homing of T cells to intestinal mucosa.

#### 3.3.2. Amino Acid Metabolites

Certain essential amino acids are produced as metabolites of probiotics. Particularly, tryptophan (Trp) is closely related to the immune system. Trp can be decomposed into various metabolites by microflora. In the gut, indolic acid derivatives, including indole-3-acetic acid (IAA), indole-3-aldehyde (IAld), indole acryloyl glycine (IAcrGly), indole lactic acid, and indole acrylic acid (IAcrA), originate from Trp catabolism. Specifically, intestinal bacteria, such as *Bacteroides*, Clostridia, and *E. coli*, can decompose Trp to tryptamine and indole pyruvic acid, which are then turned into IAA, indole propionic acid, and indole lactic acid. IAA can combine with glutamine to synthesize indolyl acetyl glutamine in the liver or converted to IAld through aerobic oxidation by peroxidase catalyzation. Indolyl propionic acid can also be further transformed to IAcrA and combine with glycine to produce IAcrGly in the liver or kidney [59]. Indole is the most effective product among various bacterial Trp metabolites. It can also attenuate TNF-*α*-induced activation of NF-*κ*B and reduce expression of the proinflammatory chemokine IL-8 as well as the adhesive capacity of pathogenic *E. coli* to HCT-8 cells [60]. In addition, both indole and its derivatives (IAld, IAA, and tryptamine) can activate intestinal innate lymphoid cells (ILCs) and regulate local IL-22 synthesis by sensitizing AhR to maintain intestinal mucosal homeostasis [61–63]. Besides, indole has been confirmed to strengthen intestinal epithelial barrier by fortifying tight junctions between cells through the pregnane X receptor (PXR) [64].

Gut commensal *Ruminococcus gnavus* and Firmicutes *C. sporogenes* have the capacity to decarboxylate Trp to tryptamine [65]. Since tryptamine exerts inhibitory effect against IDO1, it is regarded as a potential target in immune escape [66]. Skatole has been reported to inhibit CYP11A1, leading to decreased synthesis of pregnenolone, glucocorticoids, and sex steroids [67]. In the intestine, formation of endogenous steroid hormones, for instance, the anti-inflammatory glucocorticoid cortisol, is essential for the maintenance of intestinal homeostasis [68]. Therefore, skatole has been reported to play a vital role in the pathogenesis of inflammatory bowel disease (IBD).

#### 3.3.3. Bile Acids

Bile acids are mainly converted from cholesterol in hepatocytes and undergo a series of metabolic processes mediated by intestinal microflora in the intestine. With the help of probiotics, primary bile acids, namely, cholic acid and chenodeoxycholic acid, convert to deoxycholic acid and lithocholic acid, respectively [69, 70]. Since intestinal macrophages, dendritic cells, and natural killer T cells express bile acids receptors such as GPBAR1 and FXR, intestinal bile acids can bind to these receptors and suppress NLRP3 mediated inflammatory response to maintain immune homeostasis [71, 72]. In addition, bile acids also regulate chemokine CXCL16 expression on liver sinusoidal endothelial cells (LSECs) and the accumulation of CXCR6+ hepatic NKT cells, which exhibit activated phenotypes and inhibit liver tumor growth [73].

#### 3.3.4. Vitamins

Intestinal microflora has the capacity to synthesize vitamins and is their important source, especially for vitamin B [74]. As is known to all, vitamins play a vital role in regulating the immune system. Vitamin B1 is a key cofactor of tricarboxylic acid cycle. A decrease in vitamin B1 levels results in reduction of naive B cells residing in intestinal Peyer's patch, thus influencing intestinal immune function [75]. As a cofactor of sphingosine-1-phosphate (S1P) lyase, vitamin B6 is involved in the degradation of S1P. Therefore, it plays a fundamental role in maintaining S1P concentration gradient and promoting intestinal lymphocytes migration to periphery [76–80]. Besides, vitamin B also acts as a ligand for immune cells. The interaction is mediated by major histocompatibility complex MHC class I related proteins, which bind to vitamin B2, leading to the activation of mucosal-associated invariant T cells (MAITs) as well as secretion of IL-17 and IFN-*γ*. From this perspective, vitamin B2 has exerted the function of immune surveillance [81, 82].

At present, the immunoregulatory mechanism of probiotics is still not entirely clear regardless of its great variety and extensive clinical application. It requires further studies to investigate the in vivo process of probiotics through oral administration or enema therapy including the residence time, colonization, and reproduction, impact on original intestinal flora, and microbial interactions. And it is worthwhile to have a focus on the interaction of either microbiota or probiotics with immune system in regard to novel therapeutic applications. Apart from anti-TNF agents and immunomodulators, probiotics, prebiotics, and fecal microbial transplantation have been applied empirically in IBD. In addition, multiple novel strategies have already done in preclinical and clinical trials through targeting certain microbial organisms and altering mucosal immune niches. These strategies include blocking fimH to inhibit AIEC mucosal attachment, introduction of bacteriophages to eliminate pathobionts, and applying CRISPER-CAS editing to generate specific bacteriocins [83–85]. Hopefully, these approaches will be more effective which can be applied in a personalized manner in the future.

## Figures and Tables

**Table 1:** Elements of intestinal mucosal immunity.

Structures	Constitution	Effect and mechanism
Lumen	Commensal bacteria	Competitively inhibit pathogenic bacteria
		Produce antimicrobial substances
	Mucus	Traps pathogens
		Prevents access to epithelial layer
		Contains secretory immunoglobulin A
	Glycocalyx	Provides physical barrier

Epithelial layer	Enterocytes	Connected by tight junctions
		Surface TLRs induce secretion of proinflammatory chemokines, cytokines, and growth factors
		Capture some antigens
	Goblet cells	Secrete mucus
	Paneth cells	Produce defensins and antibiotic substances
	Enteroendocrine cells	Produce neuroendocrine mediators
	*γδ*iIELs	Promote *αβ*iIEL activation through cytokine and chemokine secretion
		Produce antimicrobial effectors and protect against pathogens
		Prevent inflammation-induced epithelium damage
	M cells	Capture and transport antigen

Lamina propria	*αβ*T cells, B cells, DCs, and other APCs	Initiate adaptive immune responses in lymphoid follicles
	Treg cells	Suppress activation and effector function of immune cells

**Table 2:** Mechanisms of probiotics and host interaction.

Probiotics
*Immunologic functions*
Stimulate intestinal antigen-presenting cells such as macrophages or dendritic cells and increase immunoglobulin A (IgA) secretion
Regulate lymphocyte polarization and cytokine profiles
Induce tolerance to food antigens

*Nonimmunologic functions*
Digest food and inhibitory compete with pathogens for nutrition and adhesion
Alter local PH to create an unfavorable microenvironment for pathogens
Generate bacteriocins to inhibit pathogens
Scavenge superoxide radicals
Promote epithelial antimicrobial peptides production and enhance intestinal barrier function

**Table 3:** The immunomodulatory effects of probiotics.

Literature (PMID)	Probiotic strains	Mechanism and immunologic effects
15940144, 11751960	*Lactobacillus reuteri*	Promote IL-10 secretion by Treg cells
	*Lactobacillus casei*	
17521319, 16297146	*Bifidobacterium bifidum*	Promote IL-10 secretion by mature DCs
15585777	*Lactobacillus rhamnosus*	Inhibit T-cell proliferation
		Decrease IL-2 and IL-4 secretion by mature DCs
15654823	*Bifidobacterium longum*	Promote IL-10 secretion by DCs
21740462	*E. coli* strain, Nissle 1917	Increase FoxP3+ Treg cells
19300508, 18804867	*Lactobacillus casei*, DN-114 001	Increase FoxP3+ Treg cells
		Promote IL-10 and TGF-*β* secretion
18670628	*Bifidobacterium infantis* 35, 624	Increase FoxP3+ Treg cells
		Inhibit TNF-*α* and IL-6 secretion
19029003	*Lactobacillus reuteri* (ATCC 23272)	Increase FoxP3+ Treg cells
16522473	*Bifidobacterium breve*	Activate TLR2 and promote maturation of DCs
		Increase IL-10 secretion
